# Regulatory Roles of a Novel MarR-like Protein Lmo0840 in Tetracycline-Associated Growth Phenotype and Virulence of *Listeria monocytogenes*

**DOI:** 10.3390/microorganisms14071553

**Published:** 2026-07-16

**Authors:** Yikuan Qian, Shuang Chen, Fushuang Duan, Long Wang, Lixiang Wei, Yu Yang, Xuepeng Cai, Jie Li, Qingling Meng, Jun Qiao

**Affiliations:** 1 College of Animal Science and Technology, Shihezi University, Shihezi 832003, China; 2 State Key Laboratory of Veterinary Etiological Biology, Lanzhou Veterinary Research Institute, Chinese Academy of Agricultural Sciences, Lanzhou 730046, China; 3 School of Basic Medicine, Xinjiang Second Medical University, Karamay 834000, China

**Keywords:** *Listeria monocytogenes*, multi-antibiotic resistance regulator family, tetracycline, virulence, biofilm formation

## Abstract

*Listeria monocytogenes* (*L. monocytogenes*) is a zoonotic foodborne pathogen that triggers life-threatening invasive illnesses. MarR-type transcriptional regulators widely control bacterial stress adaptation, antibiotic tolerance and virulence, playing crucial roles in the pathogenesis of diverse bacterial pathogens. Lmo0840 is a conserved member of this family; however, its physiological functions and regulatory networks in *L. monocytogenes* remain completely unexplored. To this end, we constructed an *lmo0840* deletion mutant and its complemented strain in the *L. monocytogenes* EGD-e background and systematically characterized the biological functions of Lmo0840 using phenotypic assays, transcriptomic profiling, and electrophoretic mobility shift assays (EMSA). Phenotypic characterization revealed that loss of *lmo0840* impaired growth rate under hyperosmotic, oxidative, and iron-limitation stresses, while unexpectedly promoting growth rate at 30 °C and under tetracycline stress. Consistently, Disk diffusion assays further showed that the *Δlmo0840* mutant exhibited significantly reduced inhibition zone diameters against tetracycline antibiotics compared to the EGD-e strain. Moreover, the *Δlmo0840* mutant exhibited reduced early biofilm formation and attenuated macrophage adhesion, yet showed enhanced cellular invasion and decreased virulence in a murine model. EMSA further demonstrated that Lmo0840 directly binds to the promoter region of *lmo0839* and represses its transcription, thereby contributing to bacterial adaptation to tetracycline stress. Collectively, these findings establish Lmo0840 as a pleiotropic regulator involved in environmental stress responses, tetracycline adaptation, and virulence in *L. monocytogenes*. Notably, this study identifies for the first time an Lmo0840–Lmo0839 regulatory cascade underlying tetracycline adaptation. These insights not only deepen our understanding of the molecular mechanisms governing Lm environmental adaptation and pathogenicity but also provide a potential target for the prevention and control of listeriosis.

## 1. Introduction

*Listeria monocytogenes* (*L. monocytogenes*) is a clinically important Gram-positive zoonotic pathogen that ubiquitously persists in natural environments and exhibits remarkable environmental resilience [1,2,3]. As a foodborne zoonotic bacterium, *L. monocytogenes* is capable of psychrotrophic proliferation and possesses potent biofilm formation capacity. Contamination of food and animal feed with Lm constitutes a major risk factor for listeriosis in both humans and animals [4]. Upon infection, *L. monocytogenes* penetrates intestinal epithelial barriers, disseminates to systemic tissues through lymphatic and hematogenous circulation, and crosses the blood–brain barrier and placental barrier [5,6], leading to severe systemic infections clinically presenting as meningitis, septicemia, and spontaneous abortion [7]. Currently, the first-line treatment for listeriosis depends on combination therapy using β-lactam antibiotics plus gentamicin [8]. Sulfonamides, tetracyclines, and erythromycin are recommended as alternative regimens for penicillin-allergic patients [9]. Nevertheless, the overuse and indiscriminate application of antimicrobial agents have driven the progressive emergence of antibiotic resistance in *L. monocytogenes*. The prevalence of multidrug-resistant *L. monocytogenes* strains has become a severe threat to global public health security [10,11].

Transcription factors are pivotal regulatory proteins that enable bacteria to sense and adapt to environmental stresses, thereby facilitating host colonization and in vivo survival. *L. monocytogenes* remodels genome-wide gene expression via sophisticated transcriptional regulatory networks to sustain growth and fitness under variable environmental conditions [12]. The multiple antibiotic resistance regulator (MarR) family represents a large group of conserved transcriptional regulators widely distributed across Gram-positive and Gram-negative bacteria and participates in the modulation of diverse biological processes [13]. Accumulating evidence has demonstrated that MarR family proteins govern a broad range of bacterial physiological behaviors, including antibiotic tolerance, stress resistance, and virulence factor biosynthesis, and are essential for bacterial adaptation to antimicrobial pressure and other hostile environmental cues [14,15,16,17,18]. Functional studies in multiple pathogenic bacteria have established the indispensable roles of MarR family regulators in virulence modulation. For instance, the MarR-type regulator OhrR mediates virulence expression in *Bacillus cereus* through direct and indirect regulatory mechanisms [19]. In *Staphylococcus aureus*, deletion of the MarR family gene *SarZ* decreases hemolysin production and markedly represses the transcription of virulence-related genes, consequently attenuating bacterial pathogenicity [20]. Bioinformatic analysis indicates that the protein Lmo0840 harbors a conserved winged helix–turn–helix (wHTH) motif and a canonical DNA-binding domain, which are hallmark structural features of MarR family proteins. Homology analysis was performed using the web-based NCBI BLASTP service (https://blast.ncbi.nlm.nih.gov/Blast.cgi?PROGRAM=blastp&PAGE_TYPE=BlastSearch&LINK_LOC=blasthome, accessed on 12 June 2024) against the NCBI Reference Sequence (RefSeq) database. This analysis reveals that Lmo0840 shares 32.73% amino acid sequence identity with the prototypical MarR from *Escherichia coli* (*E. coli*), indicating that Lmo0840 represents a novel MarR family homolog in *L. monocytogenes*. indicating that Lmo0840 represents a novel MarR family homolog in *L. monocytogenes*. Nevertheless, the exact biological functions of Lmo0840 remain largely uncharacterized. We therefore hypothesized that Lmo0840 modulates the transcription of downstream target genes, thereby regulating *L. monocytogenes* adaptive phenotypes under antibiotic stress and modulating its pathogenicity.

Based on the above background, the present study focused on the uncharacterized MarR family homolog Lmo0840 in *L. monocytogenes*. Using the wild-type LM EGD-e strain as the parental strain, we successfully constructed an in-frame *lmo0840* deletion mutant (*Δlmo0840*) and its complementary strain (C*Δlmo0840*). We systematically characterized the effects of Lmo0840 on environmental adaptation, biofilm formation, antibiotic stress adaption, and pathogenicity both in vitro and in vivo. By integrating RNA sequencing (RNA-seq) technology with in vitro molecular interaction assays, we further elucidated the molecular mechanism by which Lmo0840 mediates stress adaptation in *L. monocytogenes*. Collectively, this study expands the regulatory network of MarR family proteins in *L. monocytogenes* and provides solid experimental evidence and a theoretical basis for further understanding the environmental adaptation and pathogenic molecular mechanisms of this important zoonotic pathogen.

## 2. Materials and Methods

### 2.1. Bacterial Strains, Cells and Culture Conditions

*L. monocytogenes* strain EGD-e (kindly provided by Professor W. Goebel, University of Würzburg, Germany) and *E. coli* strains DH5α and BL21 were used as experimental strains. LM EGD-e was grown in brain heart infusion (BHI) broth (HOPEBIO, Qingdao, China), while *E. coli* strains were cultured in Luria–Bertani (LB) broth (HOPEBIO, Qingdao, China). Plasmids pHoss1, pHT304, and pET-28a, maintained in our laboratory, were employed for the construction of gene-mutant strains and prokaryotic protein expression vectors.

For cryopreservation, bacterial cultures were revived and grown to mid-logarithmic phase, then mixed with an equal volume of sterile 50% (*v*/*v*) glycerol (Solarbio, Beijing, China) to obtain a final concentration of 25%. The resulting suspensions were dispensed into cryogenic vials and stored at −80 °C.

Murine RAW264.7 macrophages were maintained in Dulbecco’s modified Eagle’s medium (DMEM) supplemented with 10% fetal bovine serum (FBS) (Thermo Fisher Scientific, Waltham, MA, USA) at 37 °C in a humidified incubator with 5% CO_2_.

### 2.2. Primer Design

PCR and qRT-PCR primers were designed using Primer Premier 5.0 based on the complete genome sequence of LM EGD-e (GenBank accession number NC_003210.1) (Home—Nucleotide—NCBI, https://www.ncbi.nlm.nih.gov/nuccore accessed on 12 June 2024)). The primer sequences are provided in Table 1 (conventional PCR) and Table 2 (qRT-PCR).

### 2.3. Construction and Characterization of Δlmo0840 and CΔlmo0840

Markerless deletion of the *lmo0840* gene was performed using the suicide plasmid pHoss1 according to previously described protocols [21,22]. The upstream and downstream homologous arms flanking *lmo0840* were amplified using primer pairs 0840-F1/R1 and 0840-F2/R2, respectively. The pHoss1 vector was linearized via double digestion with *Eco*R I and *Kpn* I (Takara, Shiga, Japan), and the recombinant knockout plasmid pHoss1-*Δlmo0840* was constructed by seamless cloning. The recombinant construct was electroporated into competent LM EGD-e cells, and positive deletion mutants were screened under dual selective pressure of erythromycin and elevated temperature (42 °C). For complementation, the full-length coding sequence of *lmo0840* was amplified with primers C-F and C-R. The shuttle vector pHT304 was linearized via double digestion with *Bam*H I and *Hin*d III, and the complementary plasmid pHT304-*lmo0840* was generated via seamless cloning. The recombinant plasmid was electroporated into competent *Δlmo0840* cells, and complemented strains were selected on erythromycin-containing medium. qRT-PCR was performed to compare *lmo0840* transcript levels among the wild-type, deletion, and complemented strains.

### 2.4. Effect of lmo0840 Deletion on the Growth Under Different Conditions

To compare the growth phenotypes of the three strains under various stress conditions, glycerol stocks were first revived on BHI agar and cultured overnight. Bacterial suspensions were then adjusted to a density of 10^9^ CFU/mL and inoculated at 1% (*v*/*v*) into fresh media. For temperature stress, cultures were incubated at 37 °C, 30 °C, and 42 °C, corresponding to human physiological temperature, ambient temperature, and febrile conditions, respectively. For chemical stress, cultures were inoculated into BHI broth adjusted to pH 3.0 or 9.0, or supplemented with 5% or 7.5% NaCl, 7 mM H_2_O_2_, 200 μM dipyridamole, or 0.125 μg/mL tetracycline. Growth was monitored by measuring OD_600_ every 2 h, and growth curves were generated for each condition. All experiments were performed in triplicate.

### 2.5. Assessment of the Motility in the Absence of lmo0840

Following revival and activation, the three bacterial cultures were adjusted to an OD_600_ of 0.6. One microliter of each suspension was spotted onto BHI semi-solid agar (0.3% *w*/*v*). In parallel, each strain was stabbed into tubes of BHI semi-solid medium using an inoculation loop. After incubation at 28 °C for 24 h, bacterial motility was assessed by measuring the diameter of the migration zone (motility halo) formed by each strain.

### 2.6. Effect of lmo0840 Deletion on Biofilm Formation

Single colonies of the three strains were inoculated into BHI broth and cultured overnight at 37 °C with shaking at 180 rpm. Overnight cultures were then diluted with fresh BHI broth to an OD_600_ of 0.2, and 200 μL of each standardized bacterial suspension was transferred to individual wells of a 96-well microplate. Sterile BHI broth was used as the blank control. For each strain, three biological replicates were prepared for each time point (12 h and 24 h). The plates were covered with lids and sealed with sealing tape along the edges to prevent evaporation, followed by static incubation at 37 °C for 12 h or 24 h.

After incubation, the culture supernatants were carefully aspirated to discard planktonic cells. The wells were gently washed three times with 200 μL of sterile phosphate-buffered saline (PBS), and the wash solution was completely removed after each rinse. The plates were then air-dried at room temperature for 45 min. Next, 150 μL of 0.1% (*w*/*v*) crystal violet prepared in sterile distilled water was added to each well and incubated for 30 min at room temperature. The excess stain was discarded, and the wells were washed three times with sterile PBS, followed by another 30-min air-drying step. Prior to destaining, representative wells were visualized under an inverted microscope, and images were captured to document biofilm structural morphology. The remaining crystal violet was then solubilized with 150 μL of 95% ethanol per well with gentle orbital shaking for 30 min. The absorbance at 570 nm (OD_570_) was measured using a microplate spectrophotometer to quantify the total biofilm biomass.

### 2.7. Determination of Antibiotic Sensitivity in the Absence of lmo0840

Eight antibiotics were subjected to susceptibility testing using the Kirby–Bauer (K-B) disk diffusion assay: tetracycline (30 μg/disk), ampicillin (10 μg/disk), norfloxacin (10 μg/disk), streptomycin (10 μg/disk), cefotaxime (30 μg/disk), doxycycline (30 μg/disk), ciprofloxacin (5 μg/disk), and rifampicin (5 μg/disk) (Hangzhou Microbial Reagent Co., Ltd., Zhejiang, China). The three strains were cultured overnight for activation, and bacterial suspensions were standardized to a 0.5 McFarland turbidity standard. The standardized suspensions were evenly spread onto MH agar plates with sterile cotton swabs, followed by placement of antibiotic disks onto the lawn surface. Plates were incubated statically at 37 °C for 20 h. After incubation, inhibition zones were visualized and their diameters measured using a vernier caliper. In parallel, RT-qPCR with primer pairs Q-lmo0840-F/R and Q-lmo0839-F/R was performed to quantify the relative transcript levels of *lmo0840* and *lmo0839*. All experiments were conducted in three independent biological replicates.

### 2.8. Impacts of lmo0840 Deletion on Cell Adhesion and Invasion

Murine RAW264.7 macrophages were cultured at 37 °C in a 5% CO_2_ atmosphere. LM EGD-e, LM-*Δlmo0840*, and LM-C*Δlmo0840* were grown individually to logarithmic phase (OD_600_ ≈ 0.6). Bacterial cells were then harvested, resuspended in serum-free DMEM, and adjusted to a concentration of 10^7^ CFU/mL. Macrophage monolayers were infected at a multiplicity of infection (MOI) of 10:1 (bacteria per cell). Adhesion, invasion, and intracellular proliferation were assessed for all three strains according to previously described protocols [23].

### 2.9. In Vivo Pathogenicity Assay

Sixty 6-week-old female Kunming mice were randomly divided into three groups (*n* = 6 per group): the EGD-e strain group, the *Δlmo0840* mutant group, and a sterile PBS control group. Mice received intraperitoneal injections of 0.5 mL serially diluted bacterial suspensions at concentrations ranging from 1 × 10^4^ to 1 × 10^7^ CFU/mL. Clinical signs and general health status were recorded daily, and survival curves were generated from mortality data. The 50% lethal dose (LD_50_) of each strain was calculated using Kou’s modified method [24]. A separate mouse cohort was used for bacterial load quantification and histopathological assessment. These mice were intraperitoneally inoculated with 1 × 10^6^ CFU bacteria. At day 5 post-infection, all animals were humanely euthanized via cervical dislocation. Liver and spleen tissues were aseptically collected, and bacterial burdens were quantified via plate counting. Liver, spleen, and kidney tissues were also harvested, fixed in 10% neutral-buffered formalin for 48 h, then subjected to paraffin embedding, sectioning, and hematoxylin and eosin (H&E) staining for histopathological observation.

### 2.10. Transcriptomic Sequencing

#### 2.10.1. Prokaryotic Transcriptome Sequencing Analysis

To investigate the global transcriptomic changes resulting from *lmo0840* deletion, RNA-seq was performed on the LM EGD-e strain and the *Δlmo0840* mutant. Frozen stocks stored at −80 °C were streaked onto BHI agar plates for recovery, and single colonies were inoculated into BHI broth and cultured overnight at 37 °C with shaking. The following day, cultures were subcultured and expanded, and 5 mL of bacterial culture at the exponential growth phase was collected for RNA extraction.

#### 2.10.2. RNA Extraction

Bacterial cells were harvested via centrifugation, and total RNA was extracted using the CTAB method. Residual genomic DNA was removed via DNase I treatment. RNA integrity was assessed via 1% agarose gel electrophoresis, RNA purity was determined using a NanoDrop 2000 spectrophotometer (Thermo Fisher Scientific, Wilmington, DE, USA), and RNA integrity numbers (RINs) were measured using an Agilent 2100 Bioanalyzer (Agilent Technologies, Santa Clara, CA, USA). Samples were considered suitable for library construction when they met the following criteria: total RNA ≥ 2 μg, RNA concentration ≥ 100 ng/μL, and an OD260/OD280 ratio between 1.8 and 2.2.

#### 2.10.3. Library Construction and Sequencing

rRNA was removed using the RiboCop™ rRNA Depletion Kit for Mixed Bacterial Samples (Lexogen GmbH, Vienna, Austria) to enrich mRNA, which was subsequently fragmented to an average size of approximately 300 bp. First-strand cDNA was synthesized from fragmented mRNA using random hexamer primers. During second-strand synthesis, dUTP was incorporated in place of dTTP, resulting in second-strand cDNA containing A/U/C/G bases. The resulting double-stranded cDNA possessed sticky ends, which were repaired using EndRepair Mix. Subsequently, 5′ phosphorylation and 3′ adenylation were performed to facilitate ligation of Y-shaped sequencing adapters. Prior to PCR amplification, uracil-N-glycosylase (UNG) was used to specifically digest the dUTP-containing second-strand cDNA, thereby generating a strand-specific transcriptome library that retained only the first-strand cDNA. After library quality control, paired-end 150 bp (PE150) sequencing was performed on the Illumina NovaSeq X Plus platform (Illumina Inc., San Diego, CA, USA). Three independent biological replicates were included for each strain. All samples generated at least 2.8 Gb of raw sequencing data, with raw Q20 and Q30 values of ≥99.27% and ≥96.31%, respectively. Following filtering, the base-calling error rate of the clean data was below 0.012%. Detailed sequencing quality-control statistics for all samples are provided in Supplementary Table S1.

The raw RNA sequencing FASTQ reads and normalized expression matrix supporting the findings of this study are publicly available in the NCBI Sequence Read Archive (SRA). The raw RNA-seq data have been deposited in the NCBI SRA under the BioProject accession PRJNA1492105.

#### 2.10.4. Sequencing Data Quality Control

On the Illumina sequencing platform, image signals were converted into raw sequencing data (Raw Data) in FASTQ format through CASAVA base calling. Quality-control filtering was performed according to the following criteria: removal of adapter sequences, trimming of 5′ terminal bases that were not A, G, C, or T, removal of low-quality terminal bases with quality scores below Q20, exclusion of reads containing ≥10% ambiguous nucleotides (N), and discarding reads shorter than 25 bp after trimming. High-quality reads obtained after these filtering procedures were defined as Clean Data.

#### 2.10.5. Reference Genome Alignment and Gene Quantification

Bioinformatic analyses were conducted on the Majorbio Cloud Platform (Majorbio Bio-Pharm Technology Co., Ltd., Shanghai, China) using Bowtie2 with the LM EGD-e reference genome (GenBank accession number NC_003210.1) as the alignment template. The number of valid reads mapped to each gene was quantified using featureCounts, gene expression levels were estimated using RSEM, and transcript abundance was normalized using the TPM (Transcripts Per Million) method.

#### 2.10.6. Differential Gene Expression Analysis

Differential expression analysis was performed using DESeq2 based on a negative binomial distribution model. Raw “*p*” values for individual genes were calculated and subsequently adjusted for multiple testing using the Benjamini–Hochberg (BH) procedure. The adjusted “*p*” values were reported as false discovery rates (FDRs). Differentially expressed genes (DEGs) were identified using thresholds of |log_2_ fold change| > 1 and FDR < 0.05. Functional characterization of the identified DEGs was subsequently performed through Gene Ontology (GO) annotation and Kyoto Encyclopedia of Genes and Genomes (KEGG) pathway enrichment analyses.

#### 2.10.7. Validation of Transcriptome Data via qRT-PCR

Quantitative real-time PCR (qRT-PCR) was performed to validate the expression trends of randomly selected differentially expressed genes. The 16S rRNA gene was used as the internal reference, and three independent biological replicates were included for each strain. Relative gene expression levels were calculated using the 2^−ΔΔCt^ method.

### 2.11. Electrophoretic Mobility Shift Assay (EMSA)

The coding region of *lmo0840* was amplified from LM EGD-e genomic DNA using primer pair P-F/P-R. The pET-28a vector was linearized via double digestion with *Not* I and *Bam*H I, and the recombinant expression plasmid pET-28a-*lmo0840* was constructed via seamless cloning. The plasmid was then transformed into competent *E. coli* BL21 cells, and protein expression was induced with IPTG (Solarbio, Beijing, China). The expressed protein was verified via SDS-PAGE and Western blotting, and subsequently purified via Ni-NTA affinity chromatography (Qiagen, Hilden, Germany).

In parallel, the promoter region of *lmo0839* was amplified using primers P-0839-F and P-0839-R. The purified DNA fragment was incubated with bovine serum albumin (BSA) (Sigma-Aldrich, St. Louis, MO, USA), various concentrations of recombinant Lmo0840 protein, and 5 × binding buffer at 37 °C for 30 min. Protein-DNA interactions were then analyzed via non-denaturing polyacrylamide gel electrophoresis (native PAGE) at 120 V.

### 2.12. Statistical Analysis of Data

All experimental data were analyzed and expressed as the mean ± standard deviation (SD). Statistical analyses were conducted using one-way or two-way ANOVA through GraphPad Prism 9.5 software. A value of *p* < 0.05 was considered statistically significant, *p* < 0.01 was considered highly statistically significant, and *p* > 0.05 was considered not statistically significant.

## 3. Results

### 3.1. Molecular Characteristics of the lmo0840 Gene and Its Encoded Protein

**Figure 1:**
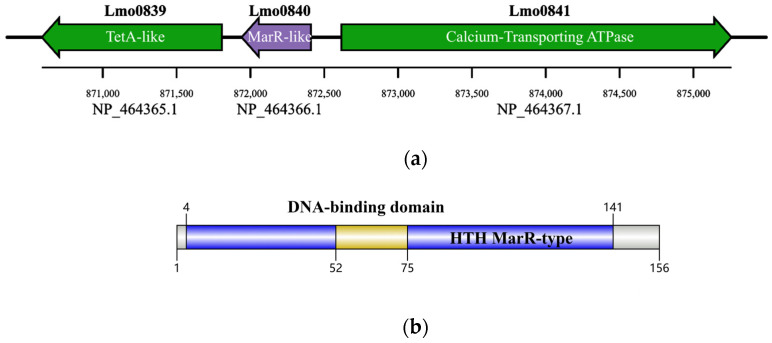
Genomic position of *lmo0840* gene and the molecular characteristics of its encoded protein in *L. monocytogenes*. (**a**) Schematic diagram of the position of the *lmo0840* gene in the *L. monocytogenes* genome; (**b**) Characteristics of the main domain in Lmo0840 protein.

Sequence analysis showed that the *lmo0840* gene (NCBI accession no. NP_464366.1) encodes a putative MarR-family transcriptional regulator, while its downstream gene *lmo0839* produces a tetracycline efflux protein classified into the major facilitator superfamily (MFS) of transporters (Figure 1a). The Lmo0840 protein consists of 156 amino acid residues. Conserved domain prediction via the ExPASy-PROSITE online database (Expasy—PROSITE, https://prosite.expasy.org/ accessed on 10 June 2024) identified a winged helix–turn–helix (wHTH) domain spanning residues 1–141 and a core DNA-binding motif at positions 52–75 (Figure 1b).

### 3.2. Construction of lmo0840-Deficient and Complementation Strains

**Figure 2:**
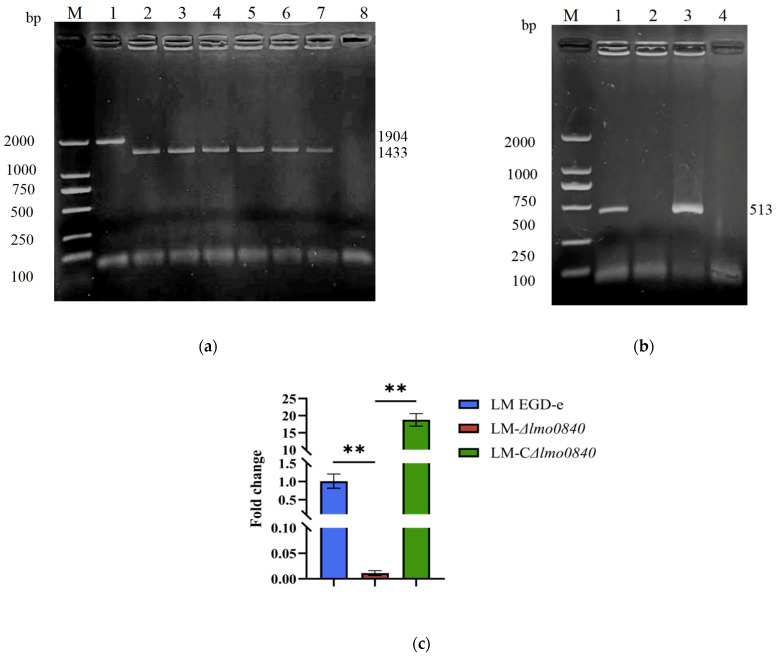
Construction and identification of *lmo0840* gene deletion and complemented strains. (**a**) Identification of the deletion strain and verification of its genetic stability. M: DNA marker DL-2000; 1: EGD-e positive control; 2–7: Identification of *Δlmo0840* in generations 5, 10, 15, 20, 25, 30; 8: negative control. (**b**) Detection of the *lmo0840* gene in the genomes of EGD-e, *Δlmo0840* and C*Δlmo0840* strains. M: DNA marker DL-2000; 1–3: PCR products of EGD-e, *Δlmo0840* and C*Δlmo0840* were amplified with C-F and C-R primers; 4: negative control. (**c**) The expression levels of *lmo0840* in EGD-e, *Δlmo0840* and C*Δlmo0840* strains were detected via qRT-PCR. (*n* = 3), ** *p* < 0.01.

PCR amplification generated a specific 1904 bp fragment for the wild-type EGD-e strain, while a 1433 bp fragment was amplified from the *Δlmo0840* deletion strain (Figure 2a). Sequencing verification confirmed successful gene fragment deletion in the *Δlmo0840* mutant (Figure 2b). qRT-PCR showed that *lmo0840* mRNA was undetectable in *Δlmo0840*, whereas abundant *lmo0840* transcripts were detected in both EGD-e and C*Δlmo0840* strains (*p* < 0.01) (Figure 2c). Collectively, these results demonstrate the successful construction of the *lmo0840* deletion and complemented strains.

### 3.3. Analysis of L. monocytogenes Growth Under Different Stress Conditions

**Figure 3:**
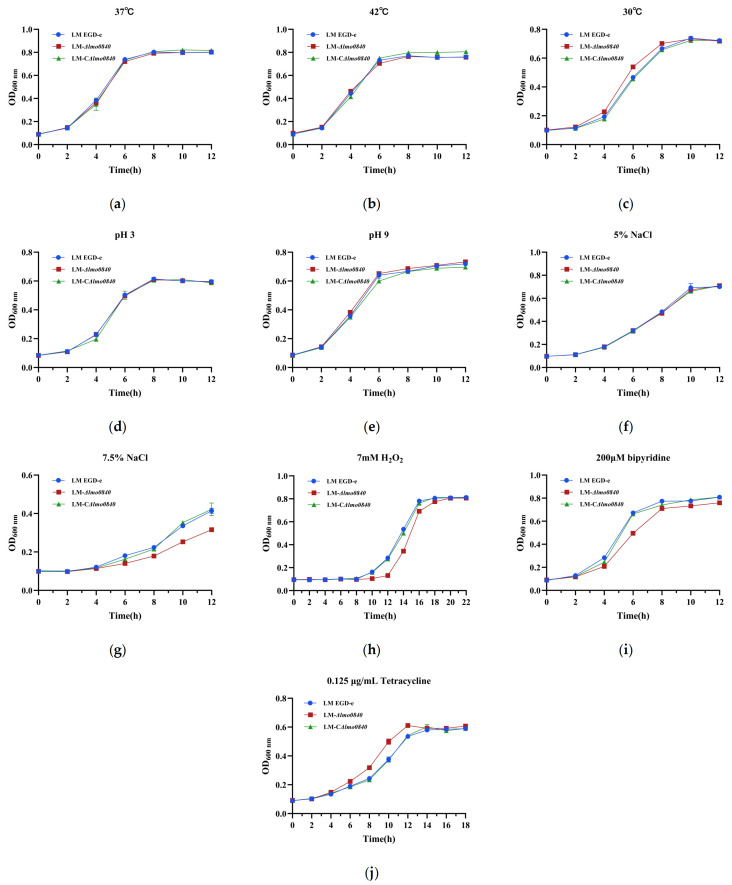
Detection of the growth of EGD-e, *Δlmo0840* and C*Δlmo0840* strains under the different conditions. (**a**–**c**) 37 °C, 42 °C, 30 °C; (**d**,**e**) pH 3.0, pH 9.0; (**f**,**g**) 5% NaCl, 7.5% NaCl; (**h**) 7 mM H_2_O_2_; (**i**) 200 μM; 2, 2′-dipyridyl; (**j**) 0.125 μg/mL tetracycline (*n* = 3).

At 30 °C, the *Δlmo0840* mutant exhibited a significantly higher growth rate than the wild-type EGD-e and complemented C*Δlmo0840* strains after 4 h of incubation, and entered the logarithmic phase earlier than the parental strain (*p* < 0.05) (Figure 3). Under 7.5% NaCl stress, growth of *Δlmo0840* was markedly inhibited after 4 h compared with EGD-e and C*Δlmo0840* (*p* < 0.05). In the presence of 7 mM H_2_O_2_, the *Δlmo0840* strain exhibited attenuated growth from 8 h (*p* < 0.05), whereas in the presence of 200 μM dipyridamole, the mutant showed reduced growth as early as 2 h (*p* < 0.05). In contrast, upon exposure to 0.125 μg/mL tetracycline, *Δlmo0840* displayed significantly enhanced growth after 4 h (*p* < 0.05). Taken together, these results suggest that *lmo0840* deletion impairs *L. monocytogenes* fitness under hyperosmotic, oxidative, and iron-limited conditions, while paradoxically conferring increased fitness to tetracycline. Overall, these findings establish Lmo0840 as a critical regulator of environmental stress adaptation in *L. monocytogenes*.

### 3.4. Deletion of lmo0840 Gene Does Not Affect L. monocytogenes Motility

**Figure 4:**
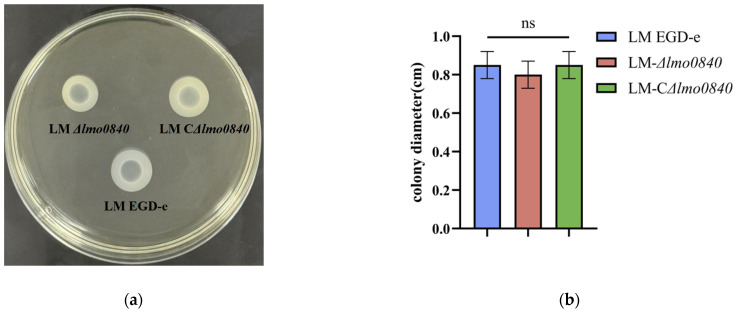
Comparison of the growth differences among EGD-e, *Δlmo0840*, and C*Δlmo0840* strains. (**a**) Diameter of the bacterial swimming ring; (**b**) Statistical analysis of swimming ring diameters. (*n* = 3). ns indicates no significant difference.

Following incubation at 28 °C for 24 h, the mean motility halo diameters for EGD-e, *Δlmo0840*, and C*Δlmo0840* were 8.5 mm, 8.0 mm, and 8.5 mm, respectively (Figure 4a). No statistically significant differences were detected among the three strains (*p* > 0.05) (Figure 4b). These findings indicate that deletion of *lmo0840* does not impair bacterial motility.

### 3.5. Deletion of the lmo0840 Gene Reduces L. monocytogenes Early Biofilm Formation Capacity

**Figure 5:**
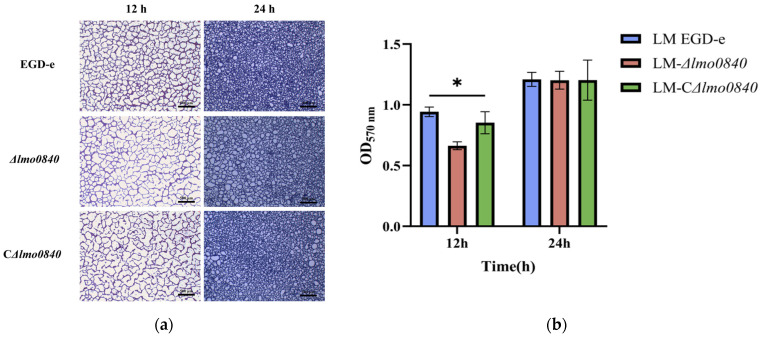
Determination of biofilm formation ability of EGD-e, *Δlmo0840* and C*Δlmo0840*. (**a**) Biofilm formation by EGD-e, *Δlmo0840*, and C*Δlmo0840* at 12 h and 24 h (200×); (**b**) OD_570 nm_ measurement of biofilms of EGD-e, *Δlmo0840*, and C*Δlmo0840* at 12 h and 24 h. (*n* = 3), * *p* < 0.05.

At 12 h post-inoculation, the biofilms formed by EGD-e and *Δlmo0840* had reached early maturation stages (Figure 5). The EGD-e biofilm displayed a relatively compact architecture, whereas the *Δlmo0840* biofilm was loosely organized and failed to form a complete structure, indicating a significantly reduced biofilm-forming capacity compared with the wild-type strain (*p* < 0.05). However, at 24 h, no statistically significant differences were observed among the three strains (*p* > 0.05). Collectively, these findings suggest that *lmo0840* is involved in the regulation of early-stage biofilm development in *L. monocytogenes*.

### 3.6. Effect of lmo0840 Gene Deletion on Antibiotic Resistance

**Figure 6:**
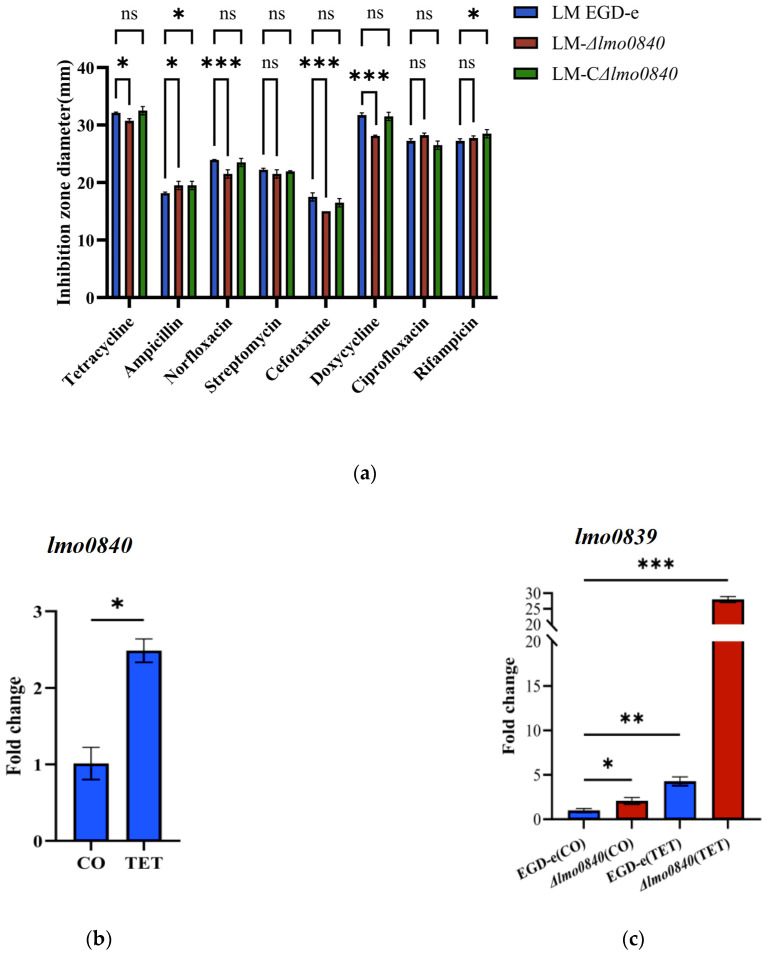
Impacts of *lmo0840* gene deletion on antibiotic sensitivity of *L. monocytogenes*. (**a**) The determination of the sensitivity to various antibiotics by the disk diffusion method; (**b**) Relative transcription of *lmo0840* was analyzed using qRT-PCR in EGD-e before (CO) and 30 min after exposure to 0.5 μg/mL tetracycline stress (TET); (**c**) Relative transcriptional levels of *lmo0839* was analyzed using qRT-PCR in EGD-e and *Δlmo0840* before (CO) and 30 min after exposure to 0.5 μg/mL tetracycline stress (TET). * *p* < 0.05, ** *p* < 0.01 and *** *p* < 0.001; ns indicates no significant difference.

Upon deletion of *lmo0840*, the *Δlmo0840* mutant exhibited significantly smaller inhibition zone diameters against tetracycline compared with the wild-type strain EGD-e (*p* < 0.05), whereas the complemented strain restored the inhibition zone size to the wild-type level. The *Δlmo0840* mutant also displayed markedly reduced inhibition zone diameters for norfloxacin, cefotaxime, and doxycycline (*p* < 0.001). No significant differences were observed in susceptibility to streptomycin or rifampicin (*p* > 0.05). These results indicate that loss of *lmo0840* markedly enhances the adaptability of *L. monocytogenes* to norfloxacin, cefotaxime, and doxycycline (Figure 6).

Under tetracycline stress at 0.5 μg/mL, the transcriptional level of *lmo0840* in EGD-e was upregulated 2.48-fold relative to the untreated control (*p* < 0.05). In the untreated *Δlmo0840* group, *lmo0839* expression was increased 2.08-fold. Following tetracycline exposure, *lmo0839* transcript levels were elevated 4.28-fold (*p* < 0.05) in EGD-e and 28-fold (*p* < 0.01) in *Δlmo0840*. These data suggest that *lmo0840* participates in the tetracycline stress response by modulating *lmo0839* expression.

### 3.7. Effects of lmo0840 Gene Deletion on Cell Adhesion, Invasion and Intracellular Proliferation

**Figure 7:**
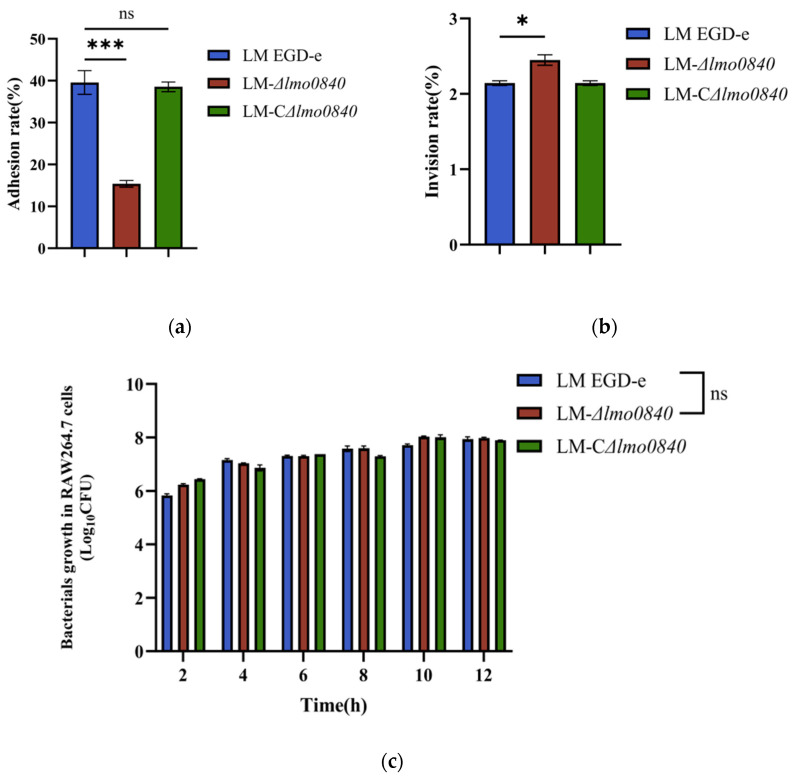
Detection of adhesion, invasion, and intracellular proliferation of EGD-e, *Δlmo0840* and C*Δlmo0840* strains in macrophages. (**a**). Invasion and adhesion rates; (**b**). Proliferation ability; (**c**). Intracellular proliferation ability. * *p* < 0.05, *** *p* < 0.001. ns indicates no significant difference.

Adhesion and invasion assays revealed that the adhesion rates of EGD-e, *Δlmo0840*, and C*Δlmo0840* to RAW264.7 macrophages were 39.57%, 15.38%, and 38.55%, respectively, while the corresponding invasion rates were 2.14%, 2.45%, and 2.09%, respectively (Figure 7a and 7b). The *Δlmo0840* mutant exhibited a significantly reduced adhesion rate compared with the wild-type and complemented strains (*p* < 0.01), yet its invasion rate was significantly elevated (*p* < 0.05). In contrast, intracellular proliferation assays showed no significant differences in bacterial replication among the three strains within macrophages (*p* > 0.05, Figure 7c). Collectively, these results indicate that *lmo0840* contributes to adhesion and invasion but is dispensable for intracellular proliferation in *L. monocytogenes*.

### 3.8. Deletion of the lmo0840 Gene Significantly Reduces the Pathogenicity of L. monocytogenes

**Figure 8:**
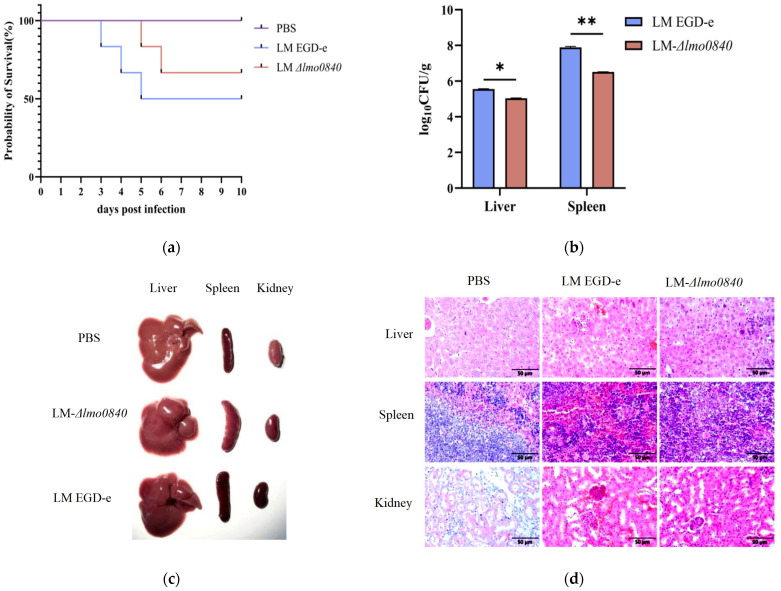
Determination of the pathogenicity of EGD-e and *Δlmo0840* in mice. (**a**) Survival curve of mice after intraperitoneal bacterial injection; (**b**) Evaluation of colonization in liver and spleen using plate count method for EGD-e and *Δlmo0840*; (**c**) Pathological changes in mouse organs caused by EGD-e and *Δlmo0840*; (**d**) Histopathological observation of liver, spleen, and kidney tissues (HE staining, scale bar: 50 μm). * *p* < 0.05, ** *p* < 0.01.

Following intraperitoneal infection with EGD-e or *Δlmo0840*, mice developed clinical signs including lethargy, anorexia, and partial eyelid closure. Mice infected with EGD-e succumbed to infection more rapidly than those infected with *Δlmo0840*, indicating that *lmo0840* deletion attenuates *L. monocytogenes* pathogenicity in vivo (Figure 8a). Organ bacterial burden analysis revealed that, compared with EGD-e, the *Δlmo0840* mutant showed a 0.5 log_10_ CFU/mL reduction in the liver and a 1.5 log_10_ CFU/mL reduction in the spleen (*p* < 0.05, Figure 8b). The LD_50_ of *Δlmo0840* was increased 3.16-fold relative to that of EGD-e (Table 3). Gross pathological findings included hepatomegaly, splenomegaly, renal congestion, and petechiae (Figure 8c). Histopathological examination revealed that, compared with the PBS control group, both the EGD-e- and *Δlmo0840*-infected groups displayed disrupted hepatic architecture with visible inflammatory cell infiltration, hepatocyte enlargement, nuclear pyknosis, and cytoplasmic dissolution. Hepatocyte necrosis was more extensive in the EGD-e group. In splenic tissues, both infection groups exhibited prominent lymphocyte accumulation. Renal tubular epithelial cells showed morphological abnormalities with interstitial congestion and edema. Overall, histopathological changes in the liver and kidneys were more pronounced in the EGD-e group than in the *Δlmo0840* group (Figure 8d).

### 3.9. Transcriptomic Analysis of EGD-e and Δlmo0840

**Figure 9:**
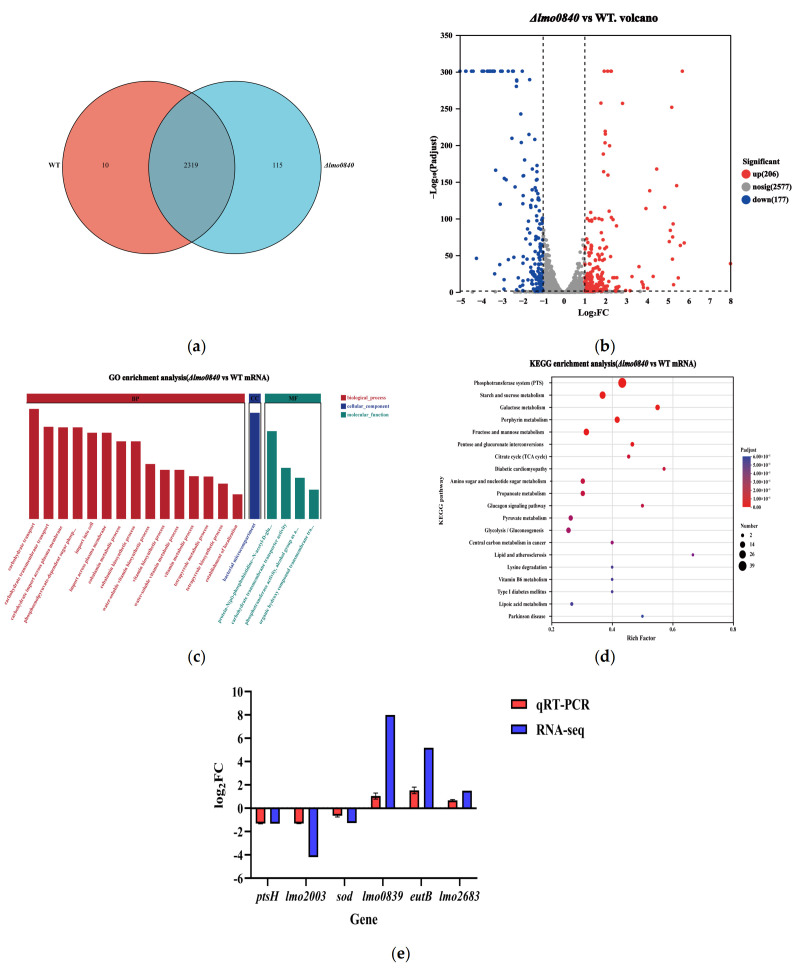
Transcriptomic analysis of EGD-e and *Δlmo0840*. (**a**) Venn diagram of co-expressed genes. (**b**) Volcano plot of differentially expressed genes between EGD-e and *Δlmo0840*; (**c**) Bar plot of GO enrichment analysis of differentially expressed genes. The labels shortened with ellipses in this panel correspond to four complete functional terms: phosphoenolpyruvate-dependent sugar phosphotransferase system; protein-N(pi)-phosphohistidine-N-acetyl-D-glucosamine phosphotransferase activity; phosphotransferase activity, alcohol group as acceptor; organic hydroxy compound transmembrane transporter activity. (**d**) Bubble plot of KEGG enrichment analysis of differentially expressed genes; (**e**) Validation of differentially expressed genes via qRT-PCR.

Transcriptomic analysis revealed 2319 co-expressed genes across all samples (Figure 9a). Differential expression analysis identified 383 genes with significantly altered transcript levels between EGD-e and *Δlmo0840* (*p* < 0.05), of which 206 were up-regulated and 177 were down-regulated in the mutant relative to the wild-type strain (Figure 9b). Among the differentially expressed genes, *lmo0839*, encoding a putative tetracycline resistance protein, exhibited the most pronounced up-regulation. Gene Ontology (GO) enrichment analysis showed that the up-regulated genes were primarily associated with ethanolamine metabolism and cobalamin biosynthesis, whereas the down-regulated genes were mainly enriched in transmembrane transport, carbohydrate catabolism, and the phosphotransferase system (PTS) (Figure 9c). KEGG pathway analysis further revealed that the differentially expressed genes were predominantly involved in metabolic pathways, including the PTS, starch and sucrose metabolism, galactose metabolism, porphyrin metabolism, and fructose and mannose metabolism (Figure 9d). The differential expression patterns were independently validated via qRT-PCR, which showed good concordance with the RNA-seq data (Figure 9e).

### 3.10. Lmo0840 Directly Binds to the Promoter of the lmo0839 Gene

**Figure 10:**
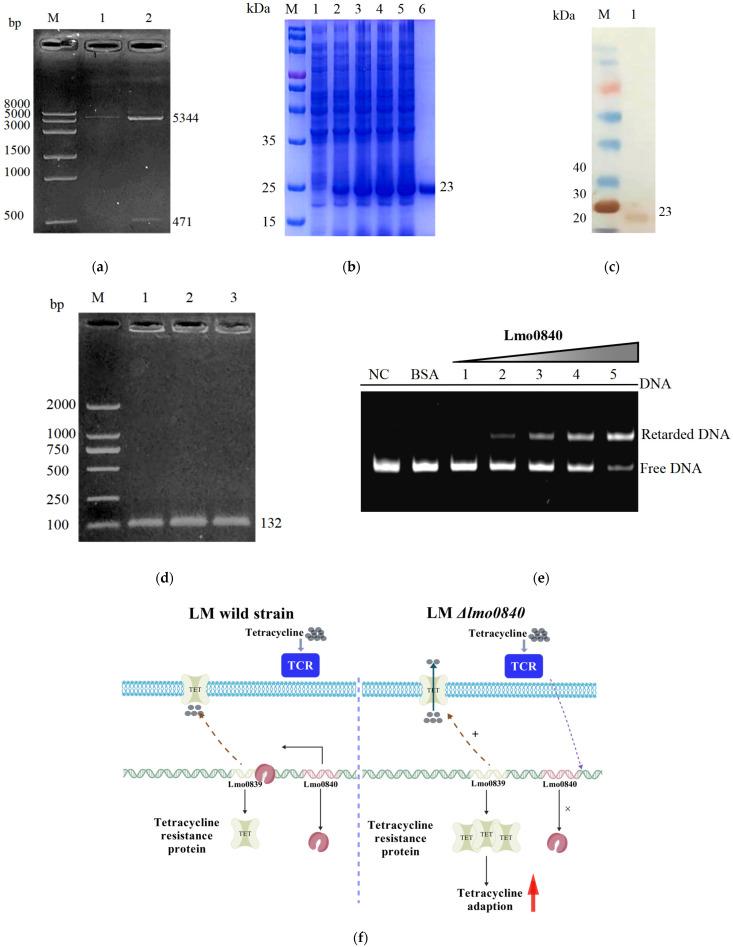
Analysis of interaction between Lmo0840 protein and the promoter of *lmo0839* gene. (**a**) Identification of recombinant plasmid pET-28a-*lmo0840* by double enzyme digestion. M: DNA marker DL-8000; 1, 2: Double-enzyme digestion of pET-28a and pET-28a-*lmo0840*. (**b**) Expression analysis of recombinant Lmo0840 protein. M: Protein molecular weight marker; 1–5: Lmo0840 protein induced for 0, 2, 4, 6, 8 h; 6: Purified Lmo0840 protein. (**c**) Western blot analysis of recombinant Lmo0840 protein. (**d**) Amplification of the *lmo0839* gene promoter region. M: DNA marker DL-2000; 1–3: PCR products of the *lmo0839* gene promoter region. (**e**) EMSA verification of the specific binding of Lmo0840 protein to the promoter of *lmo0839* gene. (**f**) Model for the regulation of tetracycline adaption by the repressor Lmo0840 via *lmo0839* in *L. monocytogenes*. ×, no expression of this gene; ↑, enhanced adaptability.

EMSAs revealed that the Lmo0840 protein binds specifically to the promoter region of *lmo0839*, which encodes the TetA efflux protein. As shown in Figure 10, increasing concentrations of Lmo0840 resulted in a progressively retarded DNA band, indicative of protein-DNA complex formation. In contrast, control lanes containing BSA or no protein showed only free DNA bands, with no shifted migration patterns (Figure 10e). Collectively, these findings demonstrate that Lmo0840 specifically interacts with the *lmo0839* promoter and thus participates in the transcriptional regulation of *lmo0839*.

## 4. Discussion

*Listeria monocytogenes* is a major foodborne pathogen capable of surviving multiple stress conditions encountered during food processing [25]. MarR-family transcriptional regulators are widely distributed among bacteria and are known to modulate antibiotic susceptibility and virulence [26,27]. In this study, structural prediction revealed that Lmo0840 contains a conserved winged helix–turn–helix (wHTH) motif and a DNA-binding domain, consistent with canonical MarR-family characteristics, although its biological function had not previously been characterized. Using homologous recombination, we constructed an *lmo0840* deletion mutant (*Δlmo0840*) and a complemented strain (C*Δlmo0840*) from the wild-type EGD-e background. To elucidate the regulatory roles of Lmo0840 in environmental adaptation and pathogenicity, we systematically assessed phenotypic traits including stress tolerance, biofilm formation, antibiotic sensitivity, and virulence.

Under carbon-limited conditions, bacteria redistribute limited energy resources to sustain survival and facilitate host colonization [28,29]. Bacterial adhesion is a prerequisite for host cell invasion [30]. Our macrophage infection assays revealed that the *Δlmo0840* mutant exhibited significantly reduced adhesion but enhanced invasion into RAW264.7 cells, while intracellular proliferation remained unaffected, suggesting that *lmo0840* primarily modulates bacterial adhesion and invasion. Transcriptomic analysis showed that 11 adhesion-associated genes were up-regulated and only three were down-regulated in the *Δlmo0840* mutant, which did not fully correlate with the observed adhesive defect. GO enrichment analysis indicated significant activation of cobalamin biosynthesis, alongside enhanced ethanolamine and 1,2-propanediol metabolic pathways. Conversely, carbon uptake systems, including the phosphotransferase system, were repressed. Cobalamin serves as an essential coenzyme that enables *L. monocytogenes* to utilize alternative carbon sources such as ethanolamine and 1,2-propanediol. The cobalamin biosynthesis pathway, together with the *eut/pdu* operons, forms the cobalamin-dependent gene cluster (CDGC) [31]. Collectively, we propose that *lmo0840* deletion remodels carbon acquisition and energy metabolism as a compensatory adaptive response. This metabolic reprogramming may in turn trigger the up-regulation of adhesion-related genes, partially offsetting the adhesive deficiency of the *Δlmo0840* mutant.

Bacterial biofilm formation is coordinately regulated by multiple factors [32]. Flagella not only confer motility but also play a critical role in the initial attachment of bacteria to abiotic and cellular surfaces [33]. Our motility assays revealed no significant difference in swimming ability between the *Δlmo0840* mutant and the wild-type EGD-e strain (Figure 4a). The *Δlmo0840* strain exhibited markedly impaired biofilm formation at 12 h post-incubation compared with EGD-e; however, this disparity disappeared at 24 h. These findings demonstrate that the early-stage biofilm defect resulting from *lmo0840* deletion is independent of bacterial motility regulation. Previous transcriptomic comparisons between biofilm and planktonic *L. monocytogenes* under nutrient limitation have identified significantly upregulated genes associated with chemotaxis, fructose and mannose metabolism, and phosphotransferase system (PTS) pathways [34], highlighting a close link between carbon metabolic homeostasis and biofilm development. In the present study, CAZyme profiling identified 14 differentially transcribed glycoside hydrolase (GH) family genes in *Δlmo0840* relative to EGD-e, including 12 downregulated and 2 upregulated transcripts. Glycoside hydrolases are essential for bacterial carbon utilization, cell wall remodeling, and host–pathogen interactions [35]; dysregulated expression of these enzymes directly alters bacterial surface properties and adhesive capacity. Previous reports have confirmed that deletion of glycoside hydrolase genes drastically attenuates pathogen adhesion to host epithelial cells [36]. Accordingly, the broad downregulation of GH genes observed here likely disrupts the balance between extracellular polysaccharide synthesis and degradation, thereby restraining early bacterial attachment and colonization. Combined with KEGG enrichment results showing widespread repression of PTS-related genes, we propose that the impaired early biofilm formation of the *Δlmo0840* mutant is collectively attributable to carbon metabolic disorder, disturbed energy homeostasis, and aberrant glycoside hydrolase expression upon *lmo0840* deletion. The precise molecular mechanism underlying this regulation remains to be elucidated in future investigations.

MarR family proteins govern virulence regulation in diverse bacterial species. In *Salmonella*, SlyA, a well-characterized MarR-type transcriptional regulator, has been shown to positively modulate bacterial pathogenicity [37]. Similarly, deletion of the MarR homolog *Aave_3922* in *Acidovorax citrulli* reduces the transcription of virulence-associated genes [38]. In the present study, transcriptomic profiling revealed widespread downregulation of phosphotransferase system (PTS) genes in the *Δlmo0840* mutant. The PTS is a central pathway for carbon acquisition and virulence control, and is indispensable for intracellular survival and full virulence of *Listeria monocytogenes* [39]. Repression of PTS signaling triggers disrupted carbon metabolism and intracellular energy imbalance, which globally attenuates bacterial virulence, even though the invasion capacity of *L. monocytogenes* is slightly elevated. We therefore hypothesize that the bacteria upregulate invasion pathways as a compensatory response to defective early adhesion and colonization. In animal infection assays, the *Δlmo0840* mutant exhibited attenuated virulence relative to the parental EGD-e strain, as evidenced by reduced bacterial loads in the liver and spleen and prolonged survival of infected mice. Moreover, the transcript levels of the PTS genes *ptsH* and *ptsI* were decreased to 0.39-fold and 0.4-fold in *Δlmo0840*, respectively. Collectively, we propose that repressed expression of PTS-related genes and impaired early adhesion capacity jointly contribute to the weakened pathogenicity of the *Δlmo0840* mutant.

Accumulating evidence has demonstrated that MarR family proteins enable bacteria to cope with various environmental stresses induced by antibiotics, organic solvents, and disinfectants [40]. In *Escherichia coli*, the MarR protein negatively regulates the *marRAB* operon, thereby promoting bacterial antibiotic resistance [41]. However, MarR family members exhibit species-specific regulatory functions across different bacterial strains, with their distinct effects determined by downstream target genes. In *Mycobacterium smegmatis*, the MarR family repressor HypS modulates resistance to hypochlorite and antibiotics by inhibiting the transcription of *hypo*, which encodes a multidrug efflux pump [42]. In *Streptococcus suis*, the MarR family repressor SatR mediates the expression of the efflux pump gene cluster *satAB*, altering bacterial susceptibility to fluoroquinolones [43]. The World Health Organization (WHO) has classified antimicrobial resistance as one of the top ten global public health threats in 2019 [44]. The widespread clinical application of antibiotics has accelerated the emergence and global transmission of multidrug-resistant bacterial strains [45]. Notably, the first multidrug-resistant *L. monocytogenes* strain isolated from pet food has been reported, indicating that resistant strains can spread through the food chain and greatly hinder the clinical treatment of foodborne bacterial diseases [46]. This situation highlights the urgent need to systematically elucidate the regulatory mechanisms underlying environmental adaptation and antibiotic stress responses in *L. monocytogenes*.

In this study, deletion of *lmo0840* markedly accelerated the growth of *L. monocytogenes* under tetracycline stress, indicating that loss of *lmo0840* enhances bacterial growth capacity under low-concentration tetracycline pressure. The *Δlmo0840* mutant exhibited a significantly smaller inhibition zone diameter against tetracycline than the wild-type EGD-e strain, and this reduction was more pronounced for doxycycline. This discrepancy may be closely associated with differences in the molecular structure and lipophilicity of the two tetracycline antibiotics. Accordingly, metabolic remodeling induced by *lmo0840* deletion may confer stronger growth adaptability under doxycycline stress, resulting in a more substantial reduction in inhibition zone size. These findings support our core conclusion that Lmo0840 participates in modulating bacterial stress responses to tetracycline antibiotics rather than mediating classical antibiotic resistance.

Transcriptomic and qRT-PCR analyses revealed significantly upregulated transcription of *lmo0839* in the *Δlmo0840* mutant relative to the EGD-e strain. Furthermore, EMSA results confirmed that the Lmo0840 protein directly binds to the promoter region of *lmo0839*. Collectively, these results suggest that Lmo0840 acts as a transcriptional repressor. By binding to the *lmo0839* promoter and inhibiting its transcription, Lmo0840 modulates the growth phenotypes of *L. monocytogenes* under tetracycline stress.

## 5. Conclusions

To date, MarR family proteins in *L. monocytogenes* exhibit diverse biological functions, yet their underlying regulatory networks remain poorly characterized. The present study elucidates the biological function of Lmo0840 and identifies the Lmo0840–Lmo0839 regulatory cascade as a potential modulator of bacterial growth adaptability under tetracycline stress. Collectively, these findings expand the current functional repertoire of the MarR family in *Listeria*, provide novel insights into the molecular interplay between environmental adaptation and pathogenicity, and offer valuable references for refining listeriosis prevention and control strategies and for exploring novel therapeutic targets.

## Figures and Tables

**Table 1:** List of primers used in this study.

Primer	Nucleotide Sequence (5→3)	Target Gene	Length (bp)	Restriction Site
*0840*-F1	CTGCAGAAGCTTCTAGAATTCCTTTGCTTAGAACCATCACGAATAA	Upstream primer for *lmo0840* gene deletion	617	*Eco*R I
*0840*-R1	ATAGGCTGCTAGAGGGTGTCATGCCCTCCTAATCT			
*0840*-F2	AGATTAGGAGGGCATGACACCCTCTAGCAGCCTAT	Downstream primer for *lmo0840* gene deletion	586	
*0840*-R2	ATCATTAGATCCCATGGTACCGCTCCTCTTGAGGTGTAATATCAGC			*Kpn *I
D-F	ATTCTCCAGTAAGCATCCCT	Verification for *lmo0840* deletion strain	1904/1433	
D-R	TGCGTCGTCTAAACTCTCAG			
C-F	GAGCTCGGTACCCGGGGATCCTTATTCAGATTTTGTTTTTAAAAC	Primers for *lmo0840* complementation construction	513	*Bam*H I
C-R	GACCATGATTACGCCAAGCTTATGGATAAAAACGAACAAGTCATGG			*Hin*d III
P-F	GCGGCCGCTTATTCAGATTTTGTTTTTAAAACTTTCTTCATCTC	Primers for pET-28a-*lmo0840* construction	485	*Not* I
P-R	GGATCCATGGATAAAAACGAACAAG			*Bam*H I

Note: The underlined sequence indicates the cleavage sites.

**Table 2:** List of primers used for real-time qPCR.

Primer Names	Nucleotide Sequence (5→3)	Product Size (bp)
*16s*-F	TCCTACGGGAGGCAGCAGT	187
*16s*-R	GGCTGCTGGCACGTAGTTA	
*eutB*-F	GGTTACCACGAAACAGCCAC	142
*eutB*-R	CATCTCCAGCACGGCTAGTT	
*sod*-F	TGCAGCACGTTTTGGTTCTG	117
*sod*-R	CCAAGAACGGGTGTTTTGCC	
*ptsH*-F	AAACAGGAATTCACGCACGC	131
*ptsH*-R	ACCAAGAGACATAACGCCCA	
Q-*lmo0840*-F	CGAACAAGTCATGGCAAACGT	92
Q-*lmo0840*-R	GTTTGTATCCCTCGAGCGCT	
Q-*lmo0839*-F	TGGTTTCAAAAGCTGCCAGC	102
Q-*lmo0839*-R	GCCCACCGATAATAGGTCCG	
*lmo2003*-F	ATGCCGAACGAAACTGCCTT	124
*lmo2003*-R	GGACGTATAGCCCAACACCG	
*lmo2683*-F	GCAGGTATGTCTACCAGCTT	105
*lmo2683*-R	GTTAGCTGCTTCTGCTTCGG	

**Table 3:** Determination of LD50 of different strains of *L. monocytogenes*.

Strain	Dose CFU	Number of Animals	Number of Deaths	Mortality Rate	LD_50_CFU
EGD-e	1.0 × 10^7^	6	6	1	10^5.53^
	1.0 × 10^6^	6	3	0.5	
	1.0 × 10^5^	6	1	0.16	
	1.0 × 10^4^	6	0	0	
*Δlmo0840*	1.0 × 10^7^	6	5	0.83	10^6.03^
	1.0 × 10^6^	6	2	0.33	
	1.0 × 10^5^	6	0	0	
	1.0 × 10^4^	6	0	0	
Control	PBS	6	0	0	-

## Data Availability

The raw data supporting the conclusions of this article will be made available by the authors without undue reservation. Data is contained within the article or Supplementary Materials.

## Supplementary Materials

The following supporting information can be downloaded at: https://www.mdpi.com/article/10.3390/microorganisms14071553/s1, Table S1: QC Statistics of EGD-e strain and *Δlmo0840* mutant.
